# Ventilation/Perfusion Mismatch in Pulmonary Vein Stenosis Secondary to Atrial Fibrillation Ablation

**DOI:** 10.22038/aojnmb.2024.79650.1561

**Published:** 2025

**Authors:** Leah Anne Christine L. Bollos, Ryosuke Kasai, Hideki Otsuka, Yoichi Otomi, Tomomi Matsuura, Tamaki Otani, Koji Yamaguchi, Takanori Bando, Yuya Ueki, Noritake Matsuda, Satoru Takashi, Shota Azane, Yamato Kunikane, Shoichiro Takao, Shusuke Yagi, Masataka Sata, Hitoshi Ikushima, Masafumi Harada

**Affiliations:** 1 Tokushima University Graduate School of Health Sciences, Tokushima City, Japan; 2 Department of Medical Imaging/Nuclear Medicine, Tokushima University Graduate School of Biomedical Sciences, Tokushima City, Japan; 3 Department of Radiology, Tokushima University Hospital, Tokushima City, Japan; 4 Department of Cardiovascular Medicine, Tokushima University Hospital, Tokushima City, Japan; 5 Advance Radiation Research, Education and Management Center, Tokushima University, Tokushima City, Japan; 6 Department of Diagnostic Radiology, Tokushima University Graduate School of Biomedical Sciences, Tokushima City, Japan; 7 Department of Radiation Oncology, Tokushima University Graduate School of Biomedical Sciences, Tokushima City, Japan

**Keywords:** Ventilation/ Perfusion Scan, Ventilation/ Perfusion Mismatch, Pulmonary Vein Stenosis, Atrial Fibrillation, Pulmonary Vein Ablation

## Abstract

We present two patients with a history of paroxysmal atrial fibrillation who developed pulmonary vein stenosis (PVS) following atrial fibrillation (AF) ablation. Case 1 involved a female patient in her 50s who was asymptomatic for pulmonary symptoms but was found to have a high degree of left superior PVS 15 months after AF ablation. This was demonstrated using contrast-enhanced computed tomography (CE-CT) and supported by findings of perfusion defects on ventilation-perfusion (V/Q) scan. Case 2 was a male patient in his 60s who developed progressive left superior PVS nine months after AF ablation, evidenced by serial CE-CT and V/Q scans.

PVS is a rare but well-known complication of pulmonary vein ablation for the treatment of AF that can lead to severe complications if left untreated. V/Q scans effectively assess the functional significance of PVS by detecting abnormal blood flow segments. Although a V/Q mismatch characterized by reduced perfusion defects is more commonly used in evaluating pulmonary embolism, PVS should not be disregarded as a differential diagnosis. Few studies emphasize the utility of V/Q scans in managing PVS and highlight V/Q mismatch as a notable finding. This case report aimed to highlight their significance.

## Introduction

Pulmonary vein stenosis (PVS) is a well-known complication of pulmonary vein ablation for treating atrial fibrillation (1). It typically manifests between 3 and 6 months post-procedure (1, 2) but can also occur later, depending on the stenosis severity and vessel involvement (2–4). PVS arises from tissue injury caused by thermal application near the pulmonary veins, resulting in luminal narrowing due to myocardial fibrosis and vessel wall thickening(5). 

 Increased lobar wedge pressure in patients with >50% stenosis may cause non-specific symptoms such as exertional dyspnea, pleuritic chest pain, cough, hemoptysis, and, in advanced stages, pulmonary hypertension (1, 6). Due to improved ablation techniques and awareness of complication risk factors, the incidence of PVS has since decreased. Severe PVS (>65% luminal narrowing) is rare, occurring in 0-3.1% of cases (4, 7). In comparison, mild to moderate cases (<50% luminal narrowing) are observed in up to 31% of patients who undergo pulmonary vein isolation (PVI) (8). Hemodynamic compensation may mask symptoms in mild-to-moderate cases (9), causing the disease to be overlooked and the true incidence of PVS to be underestimated. 

 The consensus for managing severe symptomatic PVS secondary to atrial fibrillation ablation is percutaneous intervention using pulmonary vein angioplasty with or without stent placement (10). Opinions vary in managing asymptomatic cases, with some studies suggesting the clinical benefits of early intervention (5, 10). Delayed diagnosis may lead to life-threatening progression (11), including possible progressive pulmonary vein hypoplasia proximal to the stenosis and total occlusion, which may then render percutaneous intervention ineffective (2,3). Therefore, a high index of suspicion and proper imaging screening for at-risk patients are necessary (11).

***Imaging in PVS***

 Findings on imaging modalities may be non-specific for PVS and often require anatomical and functional imaging for proper assessment. Noninvasive imaging modalities, including computed tomography (CT), magnetic resonance imaging (MRI), V/Q scan, and transesophageal echocardiography (TEE), are typically used for this purpose (1). CT angiography with a pulmonary vein protocol is considered well-suited for delineating pulmonary vein anatomy, diagnosing PVS, and planning procedures; however, it may overestimate PV occlusions and raise concerns about radiation and contrast doses, especially for serial imaging (7). 

 V/Q scans are effective for assessing the functional significance of PVS (6, 12). It can be performed using planar scintigraphy, single-photon emission computed tomography (SPECT), or SPECT combined with low-dose CT (SPECT/CT). V/Q scans are highly sensitive in detecting regions with abnormal blood flow by taking advantage of the unique pulmonary arterial segmental anatomy. Under normal conditions, the apparent blood flow in one-third of the lungs ranges from 15% to 30%. With severe PVS (>65% luminal narrowing), local perfusion within the affected pulmonary vein can substantially decrease to levels as low as 3% to 4% (13, 14). The reduced blood flow is demonstrated using radionuclide perfusion (Q) scans, widely used to evaluate relative lung perfusion among pathologies with vascular occlusions such as pulmonary embolism, neoplastic processes, or pulmonary congenital abnormalities (6). 

 During the perfusion scan (Q), the radiotracer ^99m^Tc-macroaggregated albumin (MAA) is intravenously injected and travels through the right atrium, right ventricle, and pulmonary artery, accumulating through micro-embolization in the peripheral pulmonary vascular bed. Significant occlusions lead to decreased distribution of ^99m^Tc-MAA in the affected lung areas (6). 

 V/Q mismatch characterized by reduced perfusion defects with no corresponding ventilatory abnormality is more commonly used to evaluate the likelihood of pulmonary embolism (12). In the case of PVS, V/Q mismatch can help determine the progression and functional significance of the occluded vessel in conjunction with CT or MRI findings (6, 15). Few studies emphasize the utility of V/Q scans in managing PVS or highlight V/Q mismatch as a notable finding (4, 6, 15, 16). We present two cases with such findings and highlight the role of the V/Q scan in managing patients with PVS.

## Case Reports

***Case 1***

 A female patient in her 50s with a history of paroxysmal atrial fibrillation and previous sessions of atrial fibrillation ablation returned 15 months after a recent ablation procedure with complaints of palpitations. Despite being on antiarrhythmic and calcium channel blocker medications, she had prolonged atrial fibrillation episodes, as observed on Holter monitoring. She denied shortness of breath, exertional dyspnea, hemoptysis, or chest pain throughout this time. The patient was admitted for further investigation and possible atrial fibrillation ablation.

**Figure1:**
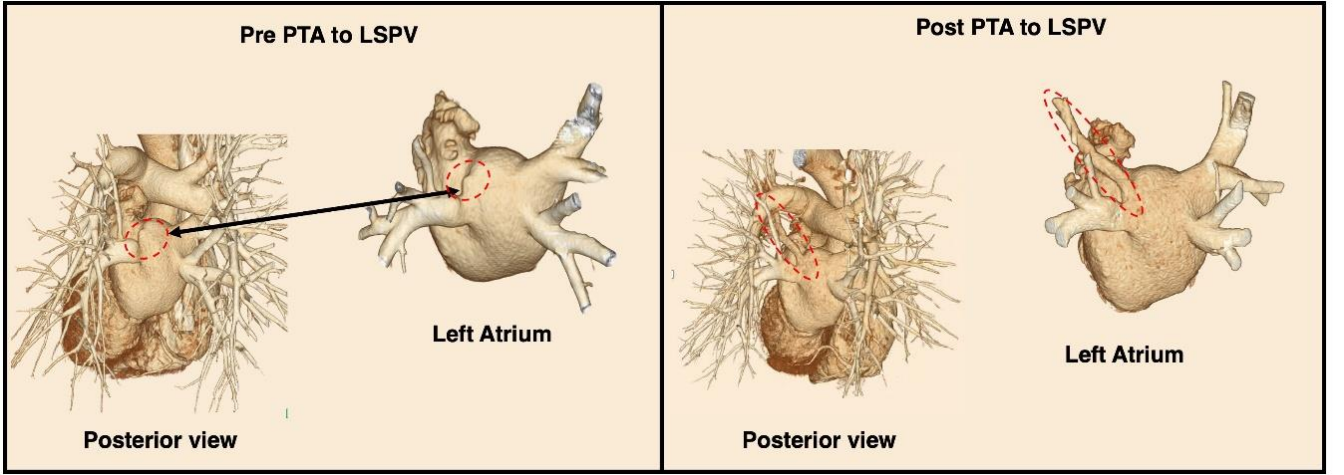
Case 1. 3-D Rendering of Pre- and Post-PTA CT Scan Images

**Figure2:**
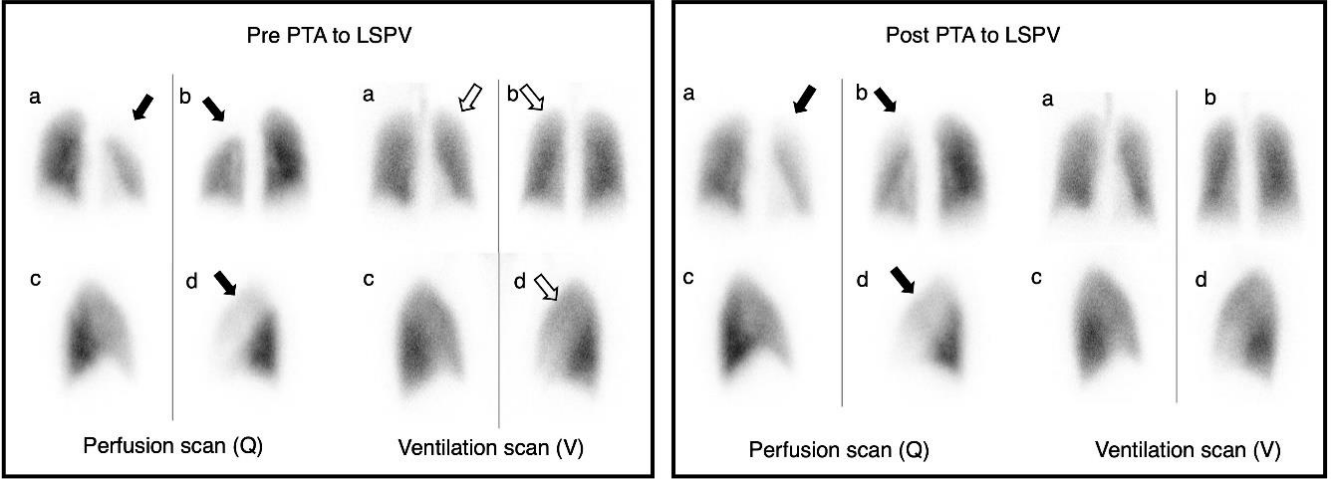
Case 1. V/Q scan, Pre- and Post- PTA to LSPV

**Figure 3:**
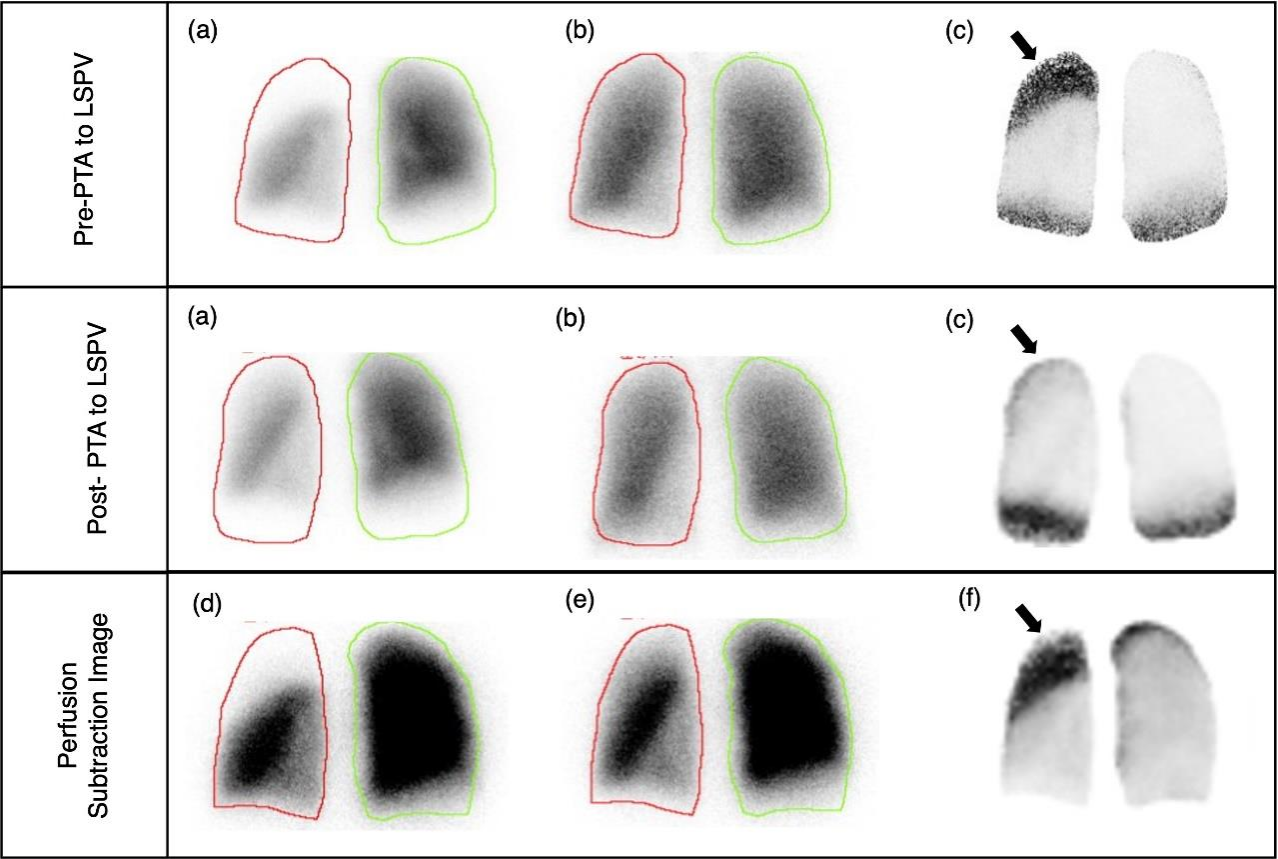
Case 1. V/Q mismatch image

 CE-CT and V/Q scans assessed morphological and functional abnormalities, respectively. Initial CT with 3D reconstruction suggested a high degree of stenosis at the LSPV inlet (Figure 1). A V/Q scan revealed normal bilateral ventilation (V) but with moderately decreased perfusion (Q) of the left upper lung (Figure 2). This V/Q mismatch defect (Figure 3) supports the CT scan findings of severe stenosis of the LSPV.

**Figure 4:**
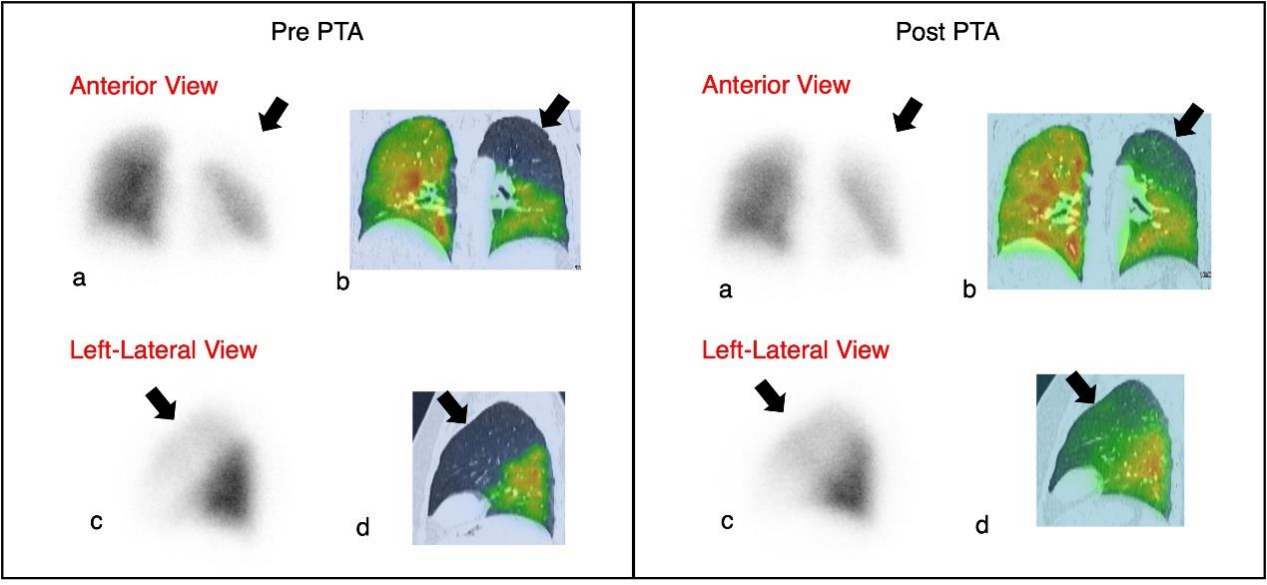
Case 1. Perfusion planar and MAA-SPECT/CT

 ICE-guided percutaneous transluminal angioplasty (PTA) was performed to dilate the LSPV. Post-procedural imaging was conducted using a hybrid SPECT/CT scanner, where lung scintigraphy was performed (Figure 4). This revealed an improved perfusion in the left upper lobe, although a mild perfusion defect was still noted. The bilateral ventilation remained normal. Consequently, the V/Q mismatch decreased; however, this was not entirely resolved (Figure 3). CT 3-D reconstruction imaging demonstrated the reestablishment of the LSPV (Figure 1).

Pre-PTA CT image shows an abrupt tapering and cut-off at the area of the left superior pulmonary vein (**outlined**), suggesting a filling defect from an occlusion. The post-PTA CT image shows the re-establishment of the LSPV (**outlined**)

Abbreviation: PTA- Percutaneous Transluminal Angioplasty

Pre-PTA V/Q scan revealed a moderately decreased perfusion (Q) of the left upper lung (**black arrows**) and normal bilateral ventilation (V). **White arrows** on the ventilation scan point toward the same area of the left upper lung with the perfusion defect

In comparison, post-PTA V/Q scan shows improved perfusion of the left upper lung with some mildly decreased perfused areas

Ventilation scan remain normal in both lungs. (**a**) Anterior view, (**b**) posterior view, (**c**) right lateral view, (**d**) left lateral view

Abbreviations: PTA- Percutaneous Transluminal Angioplasty, LSPV- Left Superior Pulmonary Vein

Planar images show a posterior view of the patient’s perfusion scan (**a**) and ventilation scan (**b**), along with the respective representation of their mismatched areas, which are darkened (**c**). A comparison with images taken post PTA shows that the mismatched areas have diminished (**arrows**), although some areas remain unresolved. This is achieved by subtracting the pre-PTA perfusion image (**d**), from the post PTA perfusion image (**e**) or (post-PTA – pre-PTA). The perfusion subtracted image illustrates the resolved mismatched areas (**f**) (**arrows**)

Abbreviation: PTA- Percutaneous Transluminal Angioplasty

The hybrid SPECT/ CT scanner enables both morphological and functional assessment of the lung. Morphological findings such as consolidation, ground glass opacity and reticular / fibrotic changes can be seen, while functional assessment using fused SPECT/CT images from any directions (axial, coronal, sagittal) provides better anatomic visualization at a higher resolution. In comparison with the planar images (**a**) & (**b**) are SPECT/CT (**c**) & (**d**) show more anatomical visualization of the lungs

Abbreviation: PTA- Percutaneous Transluminal Angioplasty

***Case 2***

**Figure 5:**
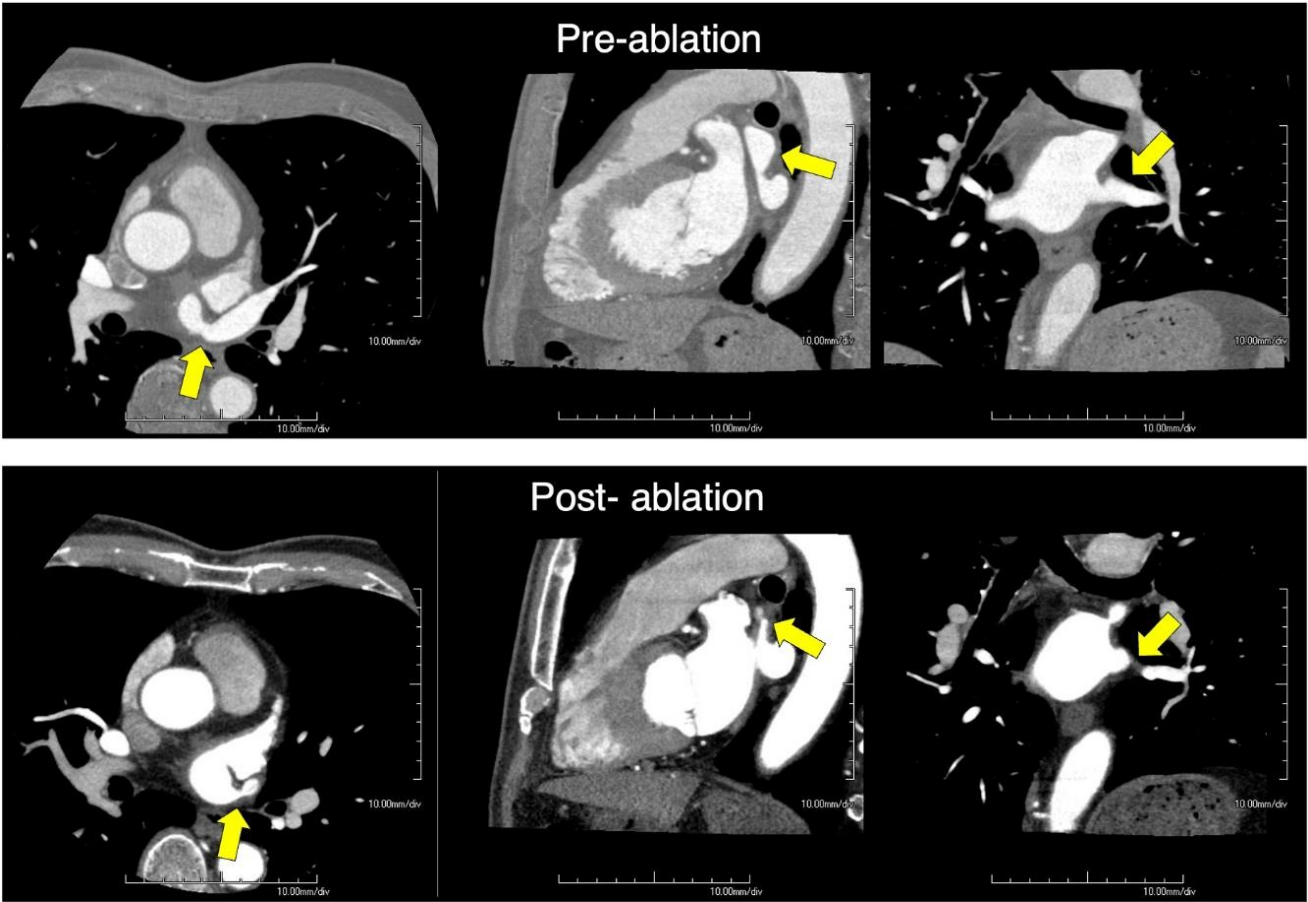
Case 2. Pre-and Post-Ablation CE-CT images

**Figure 6:**
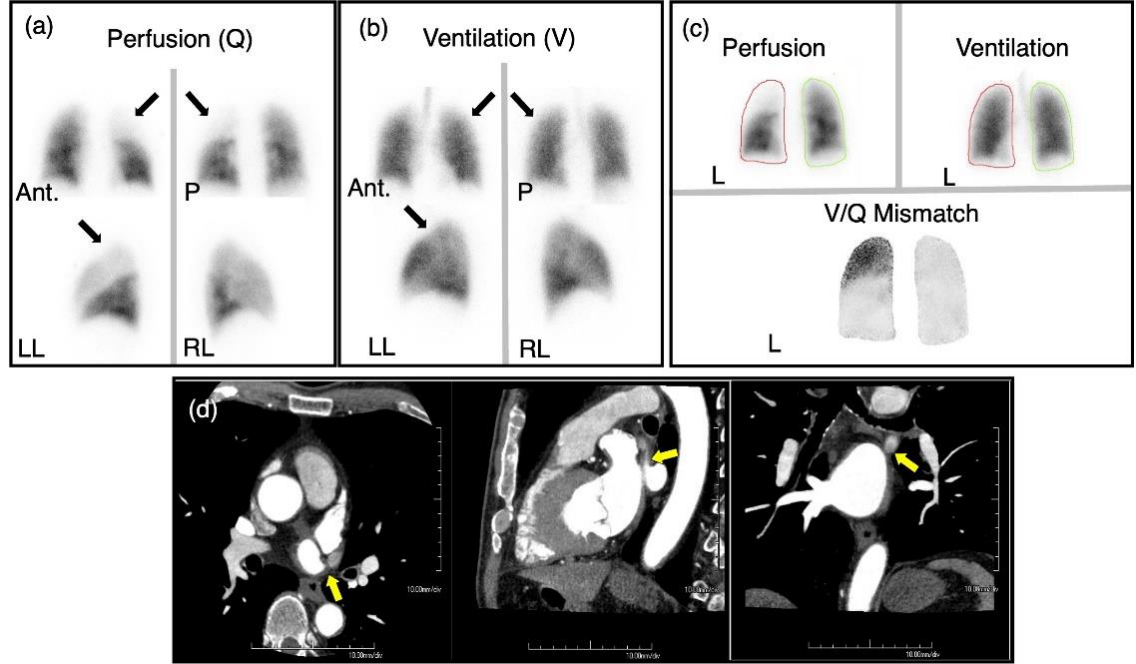
Case 2. Pre- Angioplasty Imaging

**Figure 7:**
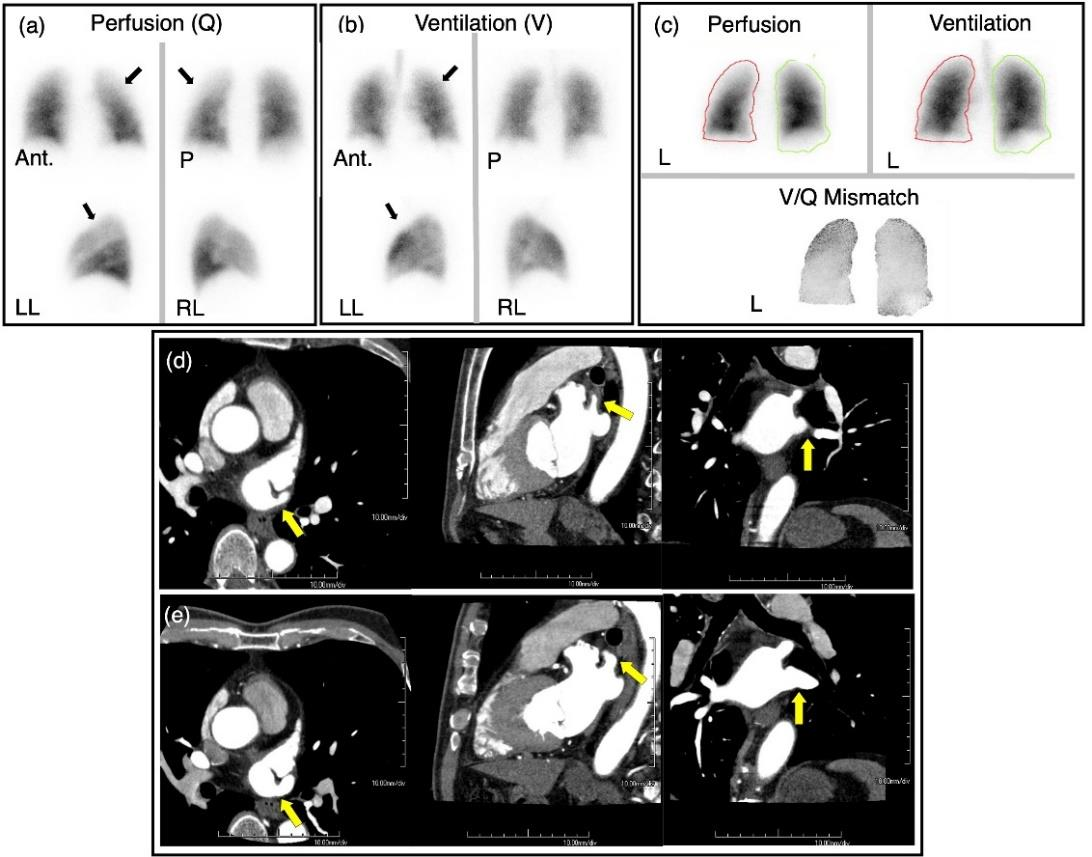
Case 2. Post- Angioplasty Images

 A male patient in his 60s with a history of recurrent paroxysmal atrial fibrillation was found to have a PVS in the left superior pulmonary vein. This was observed on a CT scan 9 months after the second ablation procedure (Figure 5). Subsequent imaging conducted 2 and 3 months later revealed a ventilation-perfusion mismatch in the left upper lobe of the lung on the V/Q scan and a worsening of the stenosis on the CE-CT scan (Figure 6). The patient did not present with any related symptoms despite imaging findings. PTA was then performed, and serial postprocedural imaging revealed a gradual improvement of lung perfusion on the affected lobe (Figure 7). On CT scan, an increase in vessel diameter and increased contrast enhancement of the left superior pulmonary vein were observed (17).

CE- CT in axial, sagittal and coronal views, taken 2 months post- ablation shows stenosis near the ostium of the LSPV (**arrows**) Comparison with pre-ablation images suggest the stenosis had developed after the procedure

Abbreviations: PTA- Percutaneous Transluminal Angioplasty, PVS- Pulmonary Vein Stenosis, CE-CT- Contrast Enhanced Computed Tomography, LSPV- Left Superior Pulmonary Vein

Perfusion (**a**) and ventilation (**b**) planar images demonstrate V/Q mismatch; Perfusion defects (**a**) are seen in the left upper lobe of the lung (**black arrows**) while corresponding areas in the ventilation scan (**b**) are preserved. V/Q mismatch (**c**) highlights the discrepancy of areas between the posterior views of perfusion and ventilation scan. Contrast-enhanced CT of the chest (**d**) in axial, sagittal and coronal views, reveal the LSPV tapering- off with minimal contrast enhancement proximal to the stenosed area noted (**yellow arrows**)

Abbreviations: PTA- Percutaneous Transluminal Angioplasty, AF- Atrial Fibrillation, PVS- Pulmonary Vein Stenosis, LSPV- Left Superior Pulmonary Vein, V/Q – Ventilation/ Perfusion, Ant. – Anterior View, L – Left, P – Posterior View, LL- Left Lateral View, RL- Right Lateral View

Perfusion (**a**) and ventilation (**b**) planar images show V/Q mismatch has diminished; Perfusion of the left upper lobe of the lung (**a**) has partially recovered while corresponding areas in the ventilation scan (**b**) remain unchanged. The mismatched areas(**c**) have also reduced. Contrast-enhanced CT of the chest (**d**) in axial, sagittal and coronal views, reveal a reduction of the stenosis with partially reestablished connections that gradually improve further on 1 year follow up (**e**)

Abbreviations: PTA- Percutaneous Transluminal Angioplasty, AF- Atrial Fibrillation, PVS- Pulmonary Vein Stenosis, LSPV- Left Superior Pulmonary Vein, V/Q – Ventilation/ Perfusion, Ant. – Anterior View, L – Left, P – Posterior View, LL- Left Lateral View, RL- Right Lateral View

## Discussion

We present two cases of PVS secondary to atrial fibrillation ablation in which CE-CT and V/Q scans were utilized for the morphological and functional assessment of PVS. Both cases exhibited a V/Q mismatch due to decreased perfusion in the upper left lung, coinciding with the CE-CT image findings.

 When significantly occluded, PVS results in a stagnation of blood flow and decreased perfusion to the part of the lung supplied by the affected vessel. This consequently depicts mismatch defects due to decreased perfusion in the areas drained by the occluded vessel. This finding is not specific to PVS and can be found in conditions with pulmonary vessel obstruction, such as pulmonary embolism, malignancies, pulmonary hypertension, and COPD-related pulmonary hypertension (9, 18). Though V/Q scans may lack specificity for PVS, some studies have noted that patients with severe PVS (>70% luminal narrowing) consistently demonstrate decreased lung perfusion and are associated with poor clinical outcomes. It is, however, less noted in those with mild stenosis (less than 50%) (3, 4). Nevertheless, when V/Q scan findings are used in the proper clinical context, PVS should be considered as a differential diagnosis. V/Q scan can also be applied quantitatively and is frequently used to assess pulmonary perfusion defects and endovascular treatment response (19). They can also aid in determining the significance of an ambiguous lesion observed on CT scans (6).

 Contrast-enhanced CT and MRI enable us to appreciate the anatomic relationship of the pulmonary veins and define the degree of anatomic stenosis by measuring vein size, allowing us to monitor and minimize any related complications (11). Post-acquisition processing from these modalities also allows 3D image reconstruction, which is beneficial for pre-procedural planning (5). In PVS, pulmonary parenchymal opacities and peripheral consolidations are typically indirect signs of significant stenosis and venous occlusion secondary to alveolar infarction or hemorrhage (15). Enhanced CT will reveal emboli as low-density structures within pulmonary arteries, and three-dimensional imaging will enable easier visualization at various angles. In our cases, no abnormal findings were observed in the lung parenchyma on the lung window CT scans.

 Transcutaneous intervention for stenosis significantly improves the perfusion to the affected lung quadrant for most patients. However, normalization typically does not occur due to the varying degrees of irreversible injury sustained by the pulmonary vasculature before stenosis relief (4). This was observed over time,in our case, using serial V/Q scans, which demonstrated partial and gradual improvement in perfusion after PTA. Based on CT findings alone, this improvement was not easily discernible in the first case.

 Despite technological improvements and enhanced ablative techniques that have reduced the incidence of PVS from AF ablation, the morbid effects of a delayed diagnosis remain a clinical concern. Prompt diagnosis of PVS requires a high degree of suspicion due to its similarity to prevalent cardiopulmonary syndromes and the potential for misdiagnosis due to misleading diagnostic tests.

 Studies have shown that using SPECT/CT has significantly improved the accuracy of lung perfusion scintigraphy (20). They are commonly applied for detecting pulmonary embolism and planning lung volume resection surgery for emphysematous and lung cancer patients. It addresses the previous limitations of V/Q planar and V/Q SPECT scans regarding anatomic and functional information. Lung perfusion scan combined with CT, performed by Hybrid SPECT/CT, has been found to offer a notably higher accuracy in detecting perfusion abnormalities compared to traditional scintigraphy techniques; however, it still lacks the necessary recommendations (20). Potential applications in PVS assessment may benefit PVS diagnostics. However, further investigation is still needed.

## Conclusions

 A V/Q mismatch in the form of reduced pulmonary perfusion is a notable finding of severe PVS and should be considered among patients with a history of atrial ablation. Along with CT, a V/Q scan is important in assessing PVS among patients with prior atrial ablation procedures. It can play a decisive role in characterizing the functional relevance of the stenosis and monitoring treatment response.
